# Temporal and Spatial Variation and Driving Forces of Soil Erosion on the Loess Plateau before and after the Implementation of the Grain-for-Green Project: A Case Study in the Yanhe River Basin, China

**DOI:** 10.3390/ijerph19148446

**Published:** 2022-07-11

**Authors:** Jiaying He, Xiaohui Jiang, Yuxin Lei, Wenjuan Cai, Junjun Zhang

**Affiliations:** 1 College of Urban and Environmental Science, Northwest University, Xi’an 710127, China; 201910212@stumail.nwu.edu.cn (J.H.); 201820851@stumail.nwu.edu.cn (Y.L.); wjuancai@stumail.nwu.edu.cn (W.C.); 202021073@stumail.nwu.edu.cn (J.Z.); 2 Department of Culture and Tourism, Yuncheng University, Yuncheng 044000, China; 3 Shaanxi Key Laboratory of Earth Surface System and Environmental Carrying Capacity, Northwest University, Xi’an 710127, China

**Keywords:** soil erosion, spatiotemporal evolution, driving forces, Grain-for-Green Project, Yanhe River Basin

## Abstract

To curb soil erosion, the Grain-for-Green Project has been implemented in the Loess Plateau region, and there have been few quantitative evaluations of the impact of ecological engineering on the spatial distribution of soil erosion on the Loess Plateau. In this paper, we used ArcGIS software, the Revised Universal Soil Loss Equation (RUSLE) model and the Geographic Detector (GeoDetector) model to investigate the changes in the spatial distribution of soil erosion and driving forces before and after the implementation of the Grain-for-Green Project in Yanhe River Basin, a typical area on the Loess Plateau. After the implementation of the Grain-for-Green Project, the soil erosion showed a decreasing trend over time and from local improvement to global optimization in space. The implementation of the Grain-for-Green Project led to changes in the dominant driving force of the spatial distribution of soil erosion, with the dominant driving force changing from the slope factor to the vegetation coverage factor. The main driving force of the two-factor interaction on soil erosion spatial differentiation changed from the slope factor and other factors to the vegetation coverage and other factors. The Grain-for-Green Project mainly influenced soil erosion by increasing the vegetation cover. The effect of the Grain-for-Green Project on the spatial distribution of soil erosion had hysteresis and spatial differences, and the direct and indirect driving forces generated by ecological engineering reached more than 50% on average.

## 1. Introduction

Soil is the part of the lithosphere that most closely connects the human–land system. Soil is not only an important material basis for maintaining the biomass of the Earth’s surface ecosystem, but also a source of raw materials for human production and living materials [1,2] (pp. 181–201) (Mabit et al., 2014; Cerdà et al., 2015). Soil erosion caused by global land use/cover change has increased by 2.5% in the last decade; the rate of soil loss is one to two orders of magnitude higher than the rate of soil formation, agricultural systems lose 75 billion tons of fertile soil per year, and soil erosion remains the dominant factor in soil degradation [3,4,5,6,7] (p. 647, pp. 20–26, pp. 11–22). Soil erosion leads to a decline in soil productivity, exacerbates human–land conflicts, threatens the richness of biological species, leads to the degradation of ecosystems, constrains agricultural production and local socioeconomic development, and further exacerbates poverty [8,9,10] (pp. 1091–1106, pp. 160–167, pp. 193–202). Global soil erosion is influenced by a combination of natural factors and human activities. Land degradation caused by human activities affects 1964.4 Mha of land globally, of which 1903 Mha has been caused by hydraulic erosion, and agricultural production has caused 75% of global soil erosion, affecting 80% of the world’s arable land and adversely affecting food production on 40% of agricultural land [11,12,13] (pp. 536–542, pp. 654–664, pp. 747–760). To curb the further deterioration of soil erosion and promote socioeconomic development and ecological protection, several soil and water conservation measures have been implemented in various countries worldwide, and meaningful results have been achieved in curbing soil erosion.

Understanding the current situation of soil erosion and revealing the driving forces and mechanisms of natural factors and human activities on soil erosion will help to further strengthen soil erosion control and promote the harmonious development of ecosystems and socioeconomic systems [14,15] (pp. 4363–4378, pp. 1032–1040). In recent years, research on soil erosion by domestic and foreign scholars has mainly focused on two aspects. One is the impact of climate and human activities on soil erosion under the background of global change [16] (pp. 773–784). Scholars believe that climate change, especially rainfall, affects soil erosion on a long-term scale [17] (pp. 1–18), whereas human activities affect soil erosion on a short-term scale, which plays an important role in soil erosion by land disturbance (such as forest destruction, tillage, and urbanization) and dam construction [18,19,20,21] (pp. 798–809, pp. 461–470, pp. 396–413). The other is the evaluation of the control effect of engineering measures (i.e., terraces and check dams), conservation tillage (i.e., farming measures, farming methods, and farming systems) and biological measures (i.e., natural forest protection and vegetation restoration) on soil erosion [22,23,24,25] (pp. 739–741, pp. 544–550, pp. 198–205, pp. 1–11). By comparing the soil erosion intensity between vegetation restoration areas and sloping farmland, it was found that soil erosion was significantly improved in seven gully vegetation restoration areas, and the temporal variation characteristics of the soil erodibility indexes were similar [26]. In terms of biological measures, studies have mainly focused on the influences of forest and grassland system cover, landscape pattern, and tree species on soil erosion, and have quantitatively evaluated the effects of vegetation coverage, effective coverage, potential coverage, critical coverage, and landscape pattern index on soil erosion [27,28,29,30,31] (pp. 0267–0275, pp. 416–417, 50–59, pp. 417–426, pp. 622–623, 140–151, pp. 505–518).

The Loess Plateau (LP) is not only an important ecological barrier in northern China, but is also one of the most severely eroded areas in China and globally [32] (pp. 579–590). The extensive soil and water loss on the LP has led to the loss of a large amount of fertile soil and a decrease in cultivated land and cultivated land yield, which have further exacerbated the local human–land conflict [33] (pp. 77–92). Silt formation from the lost sediment occurs upstream, destroying water conservancy facilities and equipment and affecting local industrial and agricultural production. The extensive soil erosion on the LP has seriously restricted the social and economic development of the local and downstream areas, and to reverse this situation, the government of the People’s Republic of China began to carry out soil and water conservation management in the 1960s. Since then, soil erosion evolution and the contributions of its driving factors have been the main concerns (such as rainfall, terraces, check dam topography, and tillage methods) on the LP [34,35] (pp. 370–378, pp. 161–170). Research has shown that soil and water conservation measures have reduced the amount of sediment into the Yellow River by 19.4 billion tons in the past 70 years, including 10.7 billion tons of sediment reduction before 1996 and 8.7 billion tons of sediment reduction in the past 20 years [36] (pp. 1–7). Studying the impacts of rainfall, vegetation, and land engineering technology on soil erosion on the LP using network and redundancy analysis (RDA), research has shown that rainfall intensity and duration were the most important factors, followed by vegetation type and land engineering technology [37] (pp. 755–764). Scholars have also determined the impact of vegetation on soil erosion in relation to the slope gradient, showing that with an increasing slope gradient, the slope overtakes vegetation cover and becomes the main control factor of soil erosion [38] (pp. 44–53). When the terrace ratio is less than 30%, the magnitude of sediment reduction is basically proportional to the terrace ratio, and when the terrace ratio is larger than 35%, the sediment reduction effect is basically stable at approximately 90% [39] (pp. 793–799). The soil erosion was further significantly reduced on the LP with the implementation of the Grain-for-Green Project (GGP) in 1999. The GGP intends to stop the cultivation of arable land with serious soil erosion, sanding, salinization, stone desertification, and low and unstable cultivated land in a planned and systematic manner, and to restore vegetation by afforestation and grass planting according to local conditions to finally achieve the goal of protecting the ecological environment. The major emphasis of the GGP policy is that of transforming slope croplands (particularly lands on steep slopes; i.e., >25°) into forestlands or grasslands on the LP [40]. During this period, scholars focused on the effect of increased forest and grass vegetation cover on soil erosion caused by ecological engineering on the LP. Since the 1960s, the implementation of soil and water conservation on the LP, including terraces, check dams, and the GGP, has resulted in a decrease of approximately 90% in the sediment load in the Yellow River [41,42] (pp. 223–243, pp. 38–41). From 1990 to 2015, the soil erosion modulus showed significantly declining trends on the LP, and land use patterns were the important factors affecting the soil erosion decline [43]. On the LP, the rainfall erosivity factor showed a tendency toward increased erosion from 2006 to 2012, whereas the vegetation coverage and management factors showed a tendency toward reduced erosion due to the implementation of the GGP. Forests and grasslands have a threshold effect in controlling soil erosion, and forests have shown relatively higher efficacy in soil conservation than grasslands [44,45] (pp. 109–116). The sediment yield coefficient, percentage of effective vegetation and erodible area were introduced to evaluate the effect of rainfall intensity on sediment yield under different vegetation conditions at the watershed scale by analyzing the impacts of different vegetation conditions on the flood sediment concentration, and sediment yield [46] (pp. 1482–1489). On the arid and semiarid LP, although large-scale vegetation restoration effectively restrained soil erosion, it also resulted in serious ecological and environmental problems. These problems mainly manifested in three ways: first, the large-scale vegetation restoration brought about an increase in the total water consumption of vegetation, drying of the deep soil layer, and a reduction in the water available for vegetation [22,47] (pp. 240–245, pp. 739–741); second, the strong transpiration of vegetation resulted in increased groundwater levels and intensified soil salinization; third, the increase in total water consumption of the vegetation led to reduced runoff and insufficient groundwater recharge [48,49,50] (pp. 1019–1022), which further aggravated the drought of the LP. Therefore, in the future, when implementing ecological engineering on the LP, we should systematically understand the effectiveness of forests and grasslands in soil erosion control under different geological conditions and geomorphologies, and eventually achieve a balance between different ecological effects (such as long-term hydrological and sedimentological changes, economic costs, and the sustainability of the affected areas).

Among the existing research results, the soil erosion changes caused by natural factors and human activities have mainly been studied from the perspective of temporal evolution. Through the control variable method, the other variables of the research year were controlled to the reference year, and the influences of the uncontrolled variables on the soil was obtained [51,52,53] (pp. 499–510, pp. 1755–1767, pp. 576–589). Due to the lack of appropriate quantitative methods, the existing studies only performed qualitative analyses on the spatial distribution of soil erosion and its driving forces. Qualitative methods have mainly been used to study the driving forces of soil erosion at a certain time point or the average condition of the driving forces within a certain time period [54,55] (pp. 31–42). However, little is known about the contribution of each natural and anthropogenic factor to the spatial distribution of soil erosion and whether they change over time. With the development of computer technology and “3S” technology, Geographic Detector (GeoDetector), a model developed by Chinese scholars to detect the spatial variability and driving forces of geographical objects (or phenomena), has provided an opportunity for the quantitative evaluation of the driving force of soil erosion spatial distribution [56,57] (pp. 428–436, pp. 250–256). This study attempted to use the Revised Universal Soil Loss Equation (RUSLE) model and the GeoDetector model to detect the dynamic evolution process of the spatial distribution and driving force of soil erosion, and to apply the local detection theory of the GeoDetector model to compensate for the shortage of global detection methods in existing studies [58,59,60] (pp. 779–790).

The Yanhe River Basin is a typical loess hilly–gully region, one of the most extensive areas of soil erosion, and one of the earliest and fastest areas to exhibit a return from farmland to forest (grass) on the LP. After the implementation of the GGP, the land use and vegetation cover increased dramatically, which was the main driving force of soil erosion reduction [61,62,63,64] (pp. 27–33, 88–94, 35–42). Little in-depth discussion has been conducted on how the spatial distribution of soil erosion changes or on the main driving forces in the Yanhe River Basin. Therefore, the main objective of this study was to evaluate the effects of the GGP on the soil erosion spatial differentiation and the collective effect of different factors on soil erosion. In the year when the Yanhe River Basin began to implement the GGP, the native system was greatly disturbed, and the newly planted artificial vegetation was in the survival period, which played a limited role in soil and water conservation and even increased the risk of erosion. Therefore, the analysis of the impact of ecological engineering on soil erosion was generally conducted by comparing before and after 2000 as the boundary. In this study, the impact of ecological engineering on the spatial distribution of soil erosion was illustrated by comparing the spatial distribution of soil erosion and the main driving forces in the Yanhe River Basin before and after the 2000s.

## 2. Materials and Methods

### 2.1. Study Area

**Figure 1:**
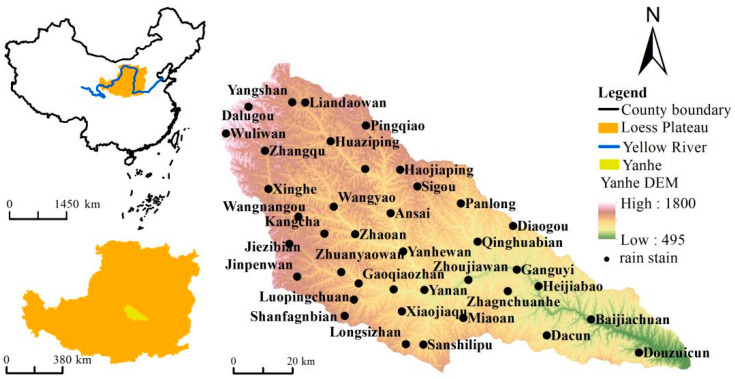
General situation of the Yanhe River Basin and distribution map of rainfall stations.

The Yanhe River Basin, a first-level tributary of the middle reaches of the Yellow River, is located in northern Shaanxi Province, China, between 36°23′ N–37°17′ N latitude and 108°45′ E–110°28′ E longitude on the Loess Plateau (Figure 1). It flows from northwest to southeast through four counties (districts) of Zhidan, Ansai, Baota, and Yanchang, with a total area of 7725 square kilometers. The basin belongs to a warm-temperate continental semi-arid climate, with a mean annual temperature of 9 °C and an annual rainfall of about 500 mm, and from northwest to southeast, the climate gradient changes significantly. The watershed landform is mainly dominated by loess hills and valleys, accounting for about 90% of the basin area, and the slope is mostly above 15°. The upstream and midstream are mainly loess beam trail hilly valley region, and the downstream is mainly loess wide-beam remnant plateau valley region [65] (pp. 1–8). The area of loess is the largest in the soil, accounting for about 80% of the cultivated area. Serious soil erosion, mainly water erosion, is mainly caused by the fragile natural conditions and the habit of long-term cultivation on steep slopes in the Yanhe River Basin [66] (pp. 569–576). According to the results of a soil erosion survey, about 60% of the Yanhe River Basin was above erosion intensity. Cultivated land, forest land, and grassland were the most dominant land use types in the Yanhe River basin, and the sum of the three accounted for more than 99% of the total area of the basin before 2010, and their area was still as high as 98.4% in 2020. They were not only the main areas of soil loss in the Yanhe River Basin, but also the main objects of ecological engineering construction. Therefore, the Yanhe River Basin in this study only included the areas of cultivated land, forest land, and grassland, and the areas of water bodies, construction land, and unused land were not included in the study areas.

### 2.2. Data Collection

The basic data of this research include daily rainfall data, land use data, *NDVI* data, *DEM* data, soil data, and socioeconomic data. Daily rainfall data from 1980 to 1990 and from 2006 to 2015 were obtained from 34 rainfall stations, provided by the Yellow River Hydrological Yearbook (Figure 1). Daily rainfall data from 1991 to 2005 was obtained from nine weather stations in and around the basin, provided by the Shaanxi Provincial Hydrological Station, Baota District Meteorological Bureau, Ansai County Meteorological Bureau, Yanchang County Meteorological Bureau, and China National Meteorological Station. The land use data in 1980s/1990s/2000s/2005s/2010s/2015s were provided by China Resources and Environment Science Data Center, with a spatial resolution of 30 m. The annual *NDVI* data from the 1980s/1990s/2000s/2005s/2010s/2015s were produced by Maximum Value Composite using the monthly average *NDVI* data of the growing season. The *NDVI* remote-sensing image data (https://ladsweb.nascom.nasa.gov/, accessed on 25 October 2021) of the *GIMMS AVHRR* in 1980s/1990s and the *MODIS* in 2000s/2005s/2010s/2015s were respectively 8 km and 250 m. The *DEM* data came from the Resource and Environmental Science Data Center of the Chinese Academy of Sciences, with a resolution of 30 m. Soil data came from the *HWSD* data set provided by the Cold and Arid Area Scientific Research Data Center. In order to improve the calculation accuracy, the data of different spatial resolutions was resampled into the 30 m × 30 m rasters by ArcGIS.

### 2.3. Research Methods

#### 2.3.1. Soil Erosion Evaluation

The modified soil loss equation (revised universal soil loss equation *RUSLE* model) by calculating the soil erosion modulus of each grid unit was used to evaluate the soil erosion in the Yanhe River Basin [67] (p. 252):

(1) A=R×K×L×S×C×P

where *A* is the annual soil erosion per unit area (t·ha^−^^1^·yr^−1^), *R* is the annual rainfall erosivity factor (MJ·mm·ha^−^^1^·h^−1^·yr^−1^), *K* is the soil erodibility factor (t·ha^−^^1^·h·ha^−^^1^·MJ^−1^·mm^−1^), *L* is the slope length factor, *S* is the slope factor, *C* is the vegetation cover and management factor, and *P* is the tillage measurement factor.

The annual rainfall erosivity factor *R* in the 1980s/1990s/2000s/2005s/2010s/2015s was calculated by rainfall erosivity model [68,69] (pp. 705–711, pp. 6–11). Firstly, the rainfall erosivity model was used to calculate the annual average rainfall erosivity of each rainfall station (meteorological station) from 1980 to 2015, and the IDW interpolation method was used to perform the spatial surface interpolation of rainfall erosivity to obtain the distribution map of annual rainfall erosivity. The calculation model of *R* is as follows:

(2) $$\begin{array}{l} R_{\mathrm{semi}\text{-}\mathrm{month}}=\alpha \sum_{k=1}^{m}{\left(P_{k}\right)}^{\beta }\\ R_{\mathrm{annum}}=\sum_{i=1}^{24}R_{\mathrm{semi}\text{-}\mathrm{monthi}}\\ \beta =0.8363+(18.177/P_{d12})+(24.455/P_{y12})\\ \alpha =21.586\beta^{-7.1891} \end{array}$$

where *k* = 1,2,…, m is the number of erosive rainfall days in a given semi-month, *P*_k_ is the erosive daily rainfall on the kth day in the semi-month, and the erosive rainfall standard used in this study is daily ≥12 mm; *P*_d12_ is the daily average of erosive rainfall in a year. *P*_y12_ is the multi-yearly average of the annual erosive rainfall. *R*_semi-month_ is the semi-monthly rainfall erosivity, and *R*_annum_ is the annual rainfall erosivity.

The soil erodibility factor *K* was calculated using the estimation method developed by Williams et al. in the erosion/productivity impact model *EPIC*. The calculation model of *K* is as follows:

(3) $$\begin{array}{l} K=\left\{0.2+0.3\exp[0.0256SAN(1-SIL/100)]\right\}\times {\left(\frac{SIL}{CLA+SIL}\right)}^{0.3}\times \\ \left[1.0-\frac{0.25C}{C+\exp(3.72-2.95C)}]\times [1.0-\frac{0.7SNI}{SNI+\exp(-5.51+22.9SNI)}\right] \end{array}$$

where *SAN*/*SIL*/*CLA* are the concentrations of *sand*/*silt*/*clay* (%), *C* is the level of total organic carbon in the soil (%), and *SNI* = 1 − *SAN*/100. *K* is converted into international units when multiplied by 0.1317 (t·ha·h·ha^−1^·MJ^−1^·mm^−1^).

We first extracted the slope *θ* and slope length *λ* according to the 30 m resolution *DEM* of the Yanhe River Basin by *ArcGIS*, and then calculated the slope factor *L* and slope length factor *S* using the calculation method established for on the LP [70,71] (pp. 1387–1396, pp. 18–23). The calculation model of *L* and *S* is as follows:

(4) $$S=\{\begin{array}{ccc} 10.8\sin\theta +0.03 & & \theta <5{}^{\circ}\\ 16.8\sin\theta -0.05 & & 5{}^{\circ}\leq \theta <14{}^{\circ}\\ 21.91\sin\theta -0.96 & & \theta \geq 14{}^{\circ} \end{array}$$

(5) $$L={\left(\lambda /22.1\right)}^{m},m=\{\begin{array}{c} 0.2\\ 0.3\\ 0.4\\ 0.5 \end{array}\begin{array}{cccc} & \theta \leq 1{}^{\circ} & & \\ & 1{}^{\circ}<\theta \leq 3{}^{\circ} & & \\ & 3{}^{\circ}<\theta \leq 5{}^{\circ} & & \\ & \theta >5{}^{\circ} & & \end{array}$$

where *S* is the slope steepness factor, *L* is the slope length factor, *θ* is the slope steepness, *λ* is the slope length, and m is the index of the slope length.

The *NDVI* is the most commonly used factor in estimating the C-factor [72] (pp. 309–324), and many researchers have established a relationship between the vegetation index and *C*. De Jong derived a formula for calculating USLE-C from the *NDVI* in 1994, but this formula cannot be used for *C* values greater than 0.431. In the process of studying the LP, researchers have established an empirical formula for calculating the *C* value from the *NDVI* [73,74] (pp. 1164–1173, pp. 9–15). The calculation model of *C* is as follows:

(6) $$C=\exp[-\alpha *NDVI*{\left(\beta -NDVI\right)}^{-1}]$$

where the values of *α* and *β* are 2 and 1, respectively.

The calculation method of the farming measure factor *P* mainly draws on the research results of the soil and water conservation factors on the LP [75,76] (pp. 1–10, pp. 1–20). The *p* value of forestland and grassland is 1 due to the lack of farming measures, and the *p* value of cultivated land is mainly assigned according to the slope (Table 1). Because farming measures are mainly used for sloping farmland, a smaller slope of sloping farmland is correlated with a smaller *p* value and a lower risk of soil erosion, and vice versa.

#### 2.3.2. Geographic Detector Model

In this study, the GeoDetector model was used to detect the spatial variability and driving force of soil erosion in the Yanhe River Basin. A geographic detector is a group of statistical methods used to detect spatial heterogeneity and reveal its driving forces. Its core idea is based on the assumption that if an independent variable has an important influence on a dependent variable, the spatial distribution of the independent variable and dependent variable should be similar [57,77,78,79] (pp. 250–256, pp. 107–127, pp. 114–115, pp. 116–134). Geographic detectors mainly include factor detection, interaction detection, ecological detection, and risk detection. In this study, two modules of factor detection and interaction detection are mainly used.

Factor detection mainly identifies the influences of the factors *X* on the spatial distribution of *Y*, which is measured by the *q* value. In this study, factor detection was used to quantitatively evaluate the influences of natural and human factors on the spatial distribution of soil erosion, and the influence of factors on the spatial distribution of soil erosion was called the driving force. A larger *q* value for factor detection is associated with a greater driving force of the factor on soil erosion spatial differentiation. *q* is calculated using the following equation:

(7) $$q=1-\left(\sum_{h=1}^{L}N_{h}\sigma_{h}^{2}\right)*{\left(N\sigma_{}^{2}\right)}^{-1}$$

where *h* = 1, …, *L* is the stratification of variable *Y* or factor *X*, which is classification or partition, *N_h_* and *N* are the number of units in layer *h* and the entire region, respectively, and *σ*^2^*_h_* and *σ*^2^ are the variances of the values in layer *h* and the entire region *Y*, respectively. The value range of *q* is from 0 to 1, and a larger value is correlated with a more obvious spatial distribution of *Y*. If the stratification is generated by the independent variable *X*, then a larger *q* value indicates a greater explanatory power of the independent variable *X* to attribute *Y*, and vice versa. In extreme cases, if the value of *q* is 1, then the factor *X* completely controls the spatial distribution of *Y*, and if the value of *q* is 0, then there is no relationship between the factor *X* and *Y*. The value of q indicates that *X* explains 100 × *q*% of *Y*.

Interaction detection mainly identifies the interactions between different risk factors *X_s_* and evaluates whether the interaction of factors *X_1_* and *X_2_* would increase or weaken the interpretation for the dependent variable *Y* by comparing *q*(*X*), *q*(*X*_1_) + *q*(*X*_2_) and *q*(*X*_1_ ∩ *X*_2_). The “interaction” implies the mutual influence of two risk factors on the explanatory power for the spatial distribution of the dependent variable *Y.* The specific operation process is as follows: first, calculate *q*(*X*_1_) and *q*(*X*_2_); second, superimpose the two layers *X*_1_ and *X*_2_ to obtain a new layer *X*_1_ ∩ *X*_2_ and calculate *q*(*X*_1_ ∩ *X*_2_); finally, judge the type of two-factor interaction according to Table 2. In this study, interaction detection was used to evaluate the combined driving of two factors on soil erosion spatial differentiation.

#### 2.3.3. Data Discretization Method

When a geographical detector is used for detection, the dependent variable *Y* is the numerical variable, and the independent variable *X* is the type variable. When the independent variable is the numerical variable, it needs to be discretized. Except for land use as the type variable, the independent variables in this study, including vegetation coverage, slope, and rainfall, were all numerical quantities that needed to be discretized. In this study, there were only three types of land use: cultivated land, forestland, and grassland. The discretization of the vegetation coverage was mainly based on the research results of the influence of vegetation coverage on soil erosion on the LP, and the vegetation coverage was divided into five classifications of 0–20%, 20–40%, 40–60%, 60–80%, and 80–100% by the equal space classification method in the ArcGIS software. The discretization of the slope was mainly based on the research results of the influence of slope on soil erosion on the LP, and the slope was divided into six categories of ≤5°, 5–10°, 10–15°, 15–20°, 20–25°, and >25° using ArcGIS. The discretization of rainfall was based on the relevant research results of erosive rainfall on the LP. In this study, the standard of daily erosive rainfall was ≥12 mm, and the moderate rainfall standard was 20–50 mm for daily rainfall, which is the main cause of erosion on the LP [76] (pp. 1–20). Therefore, the rainfall was discretized by the natural breakpoint method, which ensured that most of the intraclass rainfall difference was within 12–20 mm after classification.

## 3. Results and Analysis

### 3.1. Temporal and Spatial Variation in Soil Erosion before and after Implementation of GGP

#### 3.1.1. Temporal Variation in Soil Erosion before and after Implementation of GGP

**Figure 2:**
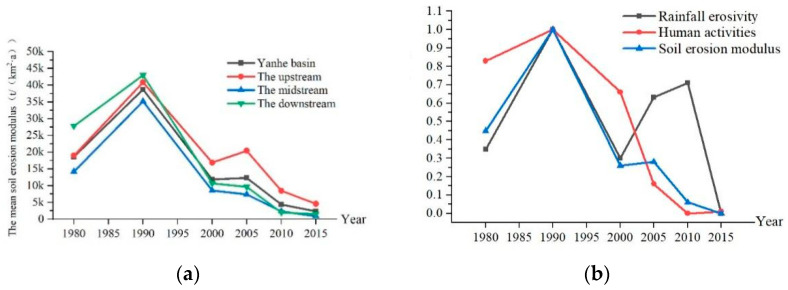
(**a**) Temporal evolution of the erosion modulus in the Yanhe River Basin. (**b**) Effects of rainfall erosivity and human activities on soil erosion intensity in the Yanhe River Basin.

Soil erosion showed a trend of first increasing and then decreasing, and especially after 2005, the soil erosion modulus continued to decrease in the Yanhe River Basin (Figure 2a). Before the implementation of the GGP (from 1980 to 2000), the mean soil erosion modulus first increased and then decreased. From 1980 to 1990, the mean soil erosion modulus rose from 18,570 t/(km^2^·a) to 38,711 t/(km^2^·a), an increase of 108%. The enhancement of soil erosion was due to the dual effects of rainfall and human activities, and human activities played a leading role in increasing the erosion (Figure 2b). From 1990 to 2000, the mean soil erosion modulus decreased to 11,848 t/(km^2^·a), a decrease of 69.4%, which was caused by rainfall and human activity, and the reduction in rainfall erosivity was greater than that in human activity. After the implementation of the GGP (from 2000 to 2015), the mean soil erosion modulus first increased and then decreased, and the soil erosion modulus continued to remain low after 2005. From 2000 to 2005, the soil erosion modulus in the entire watershed increased by 4.1%, which was caused by rainfall increasing the erosion more than human activities. From 2005 to 2015, the soil erosion modulus showed a very significant decreasing trend, and the average soil erosion modulus of the entire watershed decreased to 2303 t/(km^2^·a), a decrease of 81.3%. From 2000 to 2015, the reduction in soil erosion was mainly due to human activities, which was caused by the GGP, and the effect of rainfall alternated between increasing and decreasing erosion (Figure 2b) [41,80] (pp. 223–243, pp. 13–22).

#### 3.1.2. Spatial Variation in Soil Erosion before and after Implementation of GGP

**Figure 3:**
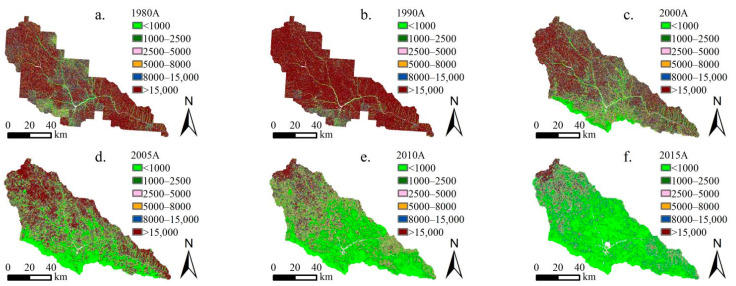
Spatial evolution of soil erosion intensity in the Yanhe River Basin from 1980 to 2015. (**a**–**f**) represent the spatial distribution of soil erosion modulus in 1980, 1990, 2000, 2005, 2010 and 2015, respectively.

**Figure 4:**
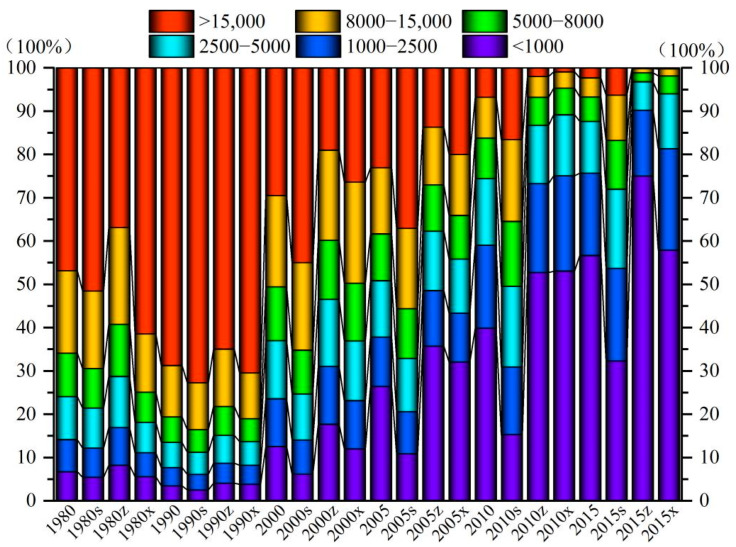
Spatiotemporal change percentage map of soil erosion intensity of the Yanhe River Basin and in the upstream, midstream, and downstream areas from 1980 to 2015; -s, -z and -x represent the upstream, midstream, and downstream areas, respectively.

According to the soil erosion classification and grading standard SL190-2007 promulgated by the Ministry of Water Resources of China, soil erosion is divided into six grades: slight, mild, moderate, strong, extremely strong, and severe. From 1980 to 2015, the spatial distribution of the soil erosion intensity showed a change trend of global deterioration–local improvement–global optimization in the Yanhe River Basin, and the spatial distinction of soil erosion showed a decreasing–increasing–decreasing trend (Figure 3 and Figure 4, units: t/(km^2^·yr)). Before the implementation of the GGP, soil erosion first showed a trend of global deterioration with the spatial differentiation slightly weakening, and then soil erosion showed a localized weakening trend with the spatial differentiation increasing. From 1980 to 1990, intense and above erosion showed a trend of spreading from upstream and downstream to the entire basin, with increases of 13.0%, 19.1%, and 5.4% in the upstream, midstream, and downstream areas, respectively. From 1990 to 2000, the moderate and below erosion shifted by intensity, and more intense erosion showed a spread from the midstream to the upstream and downstream areas, accounting for 46.5%, 37.0%, and 24.7% of these areas, respectively. The soil erosion at the southern edge of the midstream area changed significantly and was mainly microerosion. After the implementation of the GGP, the soil erosion showed a global decreasing trend from midstream–upstream–downstream, and the spatial differentiation weakened. From 2000 to 2005, the soil erosion continued to weaken from midstream to downstream, and the proportions of moderate and lower levels of midstream and downstream erosion were 62.3% and 55.9%, respectively. In the region, the soil erosion increased slightly from the midstream to the upstream areas, with intense and higher levels of erosion accounting for 67.1%, representing an increase of 10.9%. From 2005 to 2015, the soil erosion showed a trend of continuous weakening from midstream–downstream–upstream and basically achieved global optimization in 2015, when slight, mild, moderate, strong, very strong, and severe erosion accounted for 56.7%, 19.0%, 1.9%, 5.7%, 4.4%, and 2.3% of the total watershed area, 75.0%, 15.2%, 6.6%, 2.1%, 0.9%, and 0.2% of the midstream area, 57.9%, 23.4%, 12.8%, 4.1%, 1.6%, and 0.2% of the downstream area, and 32.3%, 21.4%, 18.3%, 11.3%, 10.5%, and 6.3% of the upstream area, respectively. In conclusion, the gradual implementation of the GGP in the midstream, downstream, and upstream areas of the Yanhe River Basin led to a gradual weakening of the soil erosion from midstream–downstream–upstream.

### 3.2. Detection of Spatial Distribution Drivers of Soil Erosion in Yanhe River Basin

**Figure 5:**
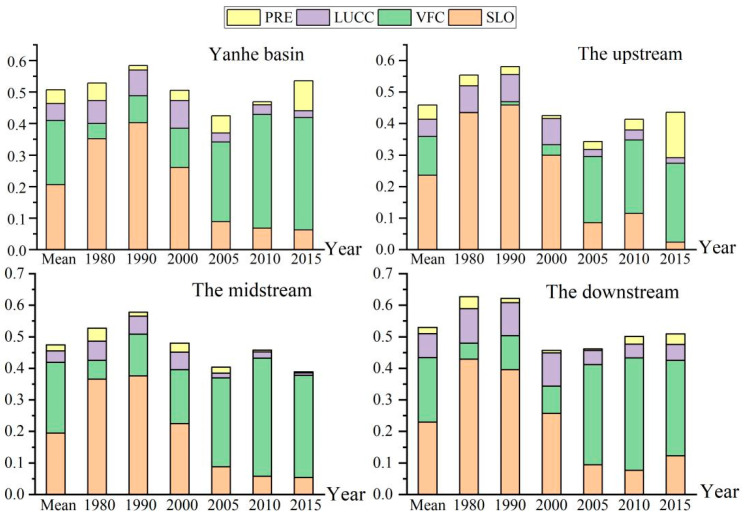
Spatiotemporal variation in driving forces for the spatial differentiation of soil erosion in the Yanhe River Basin and the upstream, midstream, and downstream areas from 1980 to 2015; PRE, LUCC, VFC, and SLO are the driving forces of rainfall, land use, vegetation coverage, and slope, respectively.

Existing studies have shown that slope, rainfall, land use type and vegetation coverage are the key factors affecting soil erosion [81,82] (pp. 1321–1334, pp. 673–686). Therefore, this study attempted to investigate the effects of slope, rainfall, land use type, and vegetation cover on the spatial distribution of soil erosion in the Yanhe River Basin. GeoDetector detection showed that there was a significant driving effect (*p* < 0.001) on the spatial variability of soil erosion by the slope, vegetation coverage, land use type, and rainfall in the Yanhe River Basin from 1980 to 2015. During this time period, the average driving force of the spatial distribution of soil erosion in the Yanhe River Basin was as follows: slope factor (0.206) > vegetation coverage factor (0.204) > land use factor (0.054) > rainfall factor (0.043). The driving force of the slope factor was slightly greater than that of the vegetation coverage factor, and the driving force of the slope factor was 3.8 times and 4.7 times that of the land use factor and rainfall factor, respectively (Figure 5).

#### 3.2.1. Temporal Evolution of Driving Force

The driving forces of the slope, vegetation cover, land use type, and rainfall factors exhibited different trends over time from 1980 to 2015 (Figure 5). The driving force of the slope factor first increased and then decreased, with an overall decreasing trend. It increased from 0.352 in 1980 to 0.403 in 1990 and continued to decrease to only 0.063 in 2015, a decrease of 84.4%. The driving force of the vegetation coverage factor continuously increased, from 0.048 in 1980 to 0.356 in 2015, an increase of 6.4 times. The driving force of the land use factors first increased and then decreased, showing an overall decreasing trend. It increased by 20.5% from 1980 to 2000 and decreased by 75% from 2000 to 2015, and the driving force in 2015 was only 30.1% of that in 1980. The driving force of the rainfall factor increased and decreased alternately, with an overall increasing trend. In conclusion, among the driving factors of the spatial differentiation of soil erosion, the driving forces of the slope and land use factors showed an increasing trend, and the driving forces of vegetation cover and rainfall factors showed a weakening trend. The variation in the driving force of the vegetation cover was larger than that of the other three factors, so the driving force of the spatial variation in soil erosion was vegetation cover factor > rainfall factor > slope factor > land use factor in 2015.

#### 3.2.2. Spatial Variation in Driving Forces

From 1980 to 2015, the driving forces of the slope factor, vegetation coverage factor, land use factor, and rainfall factor exhibited spatial differentiation, and the factors playing the dominant driving forces differed in the upper, middle, and lower reaches (Figure 5). In the upstream region, the slope factor was the dominant driver, with a value of 0.236, and the drivers of the vegetation cover factor, land use factor, and rainfall factor were 52.0%, 23.1%, and 19.2%, respectively. In the midstream region, vegetation cover was the dominant driver with a value of 0.224, and the slope factor, land use factor, and rainfall factor drivers were 86.9%, 16.1%, and 8.3% of that value, respectively. In the downstream area, the slope factor was the dominant driver with a value of 0.230, and the vegetation cover factor, land use factor, and rainfall factor drivers were 88.7%, 33.3%, and 8.8% of the slope factor driver, respectively. The spatial differentiation of soil erosion in the Yanhe River Basin is mainly determined by its geological and geomorphological conditions. The gully densities in the middle and upper reaches of the Yanhe River Basin are higher than that downstream, which easily causes erosion, and the slope downstream is smaller than those in the upper and middle reaches, which are valley landforms. However, the vegetation coverage in the middle reaches is higher than that in the downstream and upstream reaches. Therefore, the driving force of the slope factor was upstream > downstream > midstream, and the driving force of the vegetation cover factor was midstream > downstream > upstream.

### 3.3. Changes in Driving Forces of Spatial Distribution of Soil Erosion before and after Implementation of GGP

#### 3.3.1. Changes in Dominant Driving Force before and after Implementation of GGP

**Figure 6:**
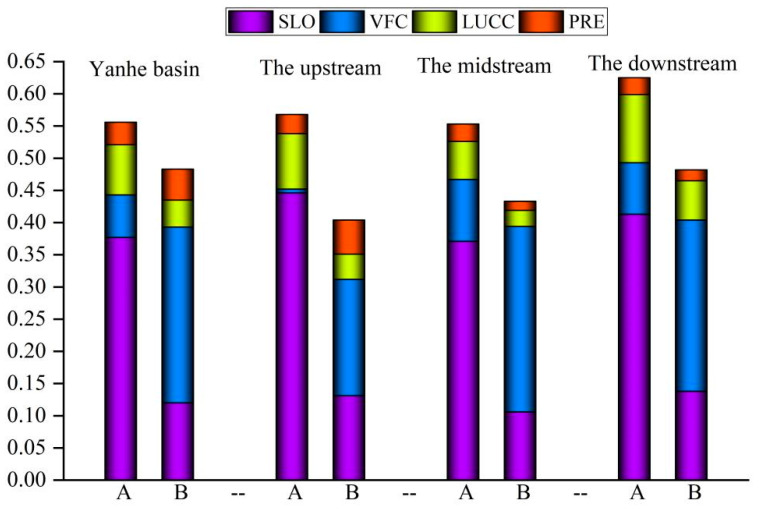
Driving forces of the spatial differentiation of soil erosion before and after the implementation of the GGP; PRE, LUCC, VFC, and SLO; A is the driving force before 2000, and B is that after 2000.

**Figure 7:**
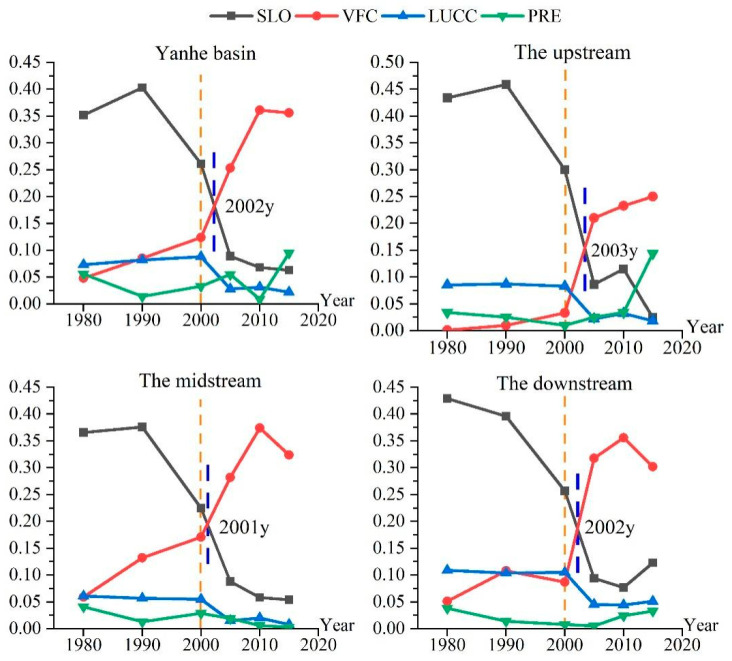
Temporal evolution of the driving forces for the spatial differentiation of soil erosion.

The results showed that the dominant driving force of the spatial differentiation of soil erosion changed significantly before and after the implementation of the GGP. Before the implementation of the GGP, the average driving force was as follows: slope factor (0.377) > land use factor (0.079) > vegetation coverage factor (0.066) > rainfall factor (0.035). After the implementation of the project, the average driving force was as follows: vegetation coverage factor (0.273) > slope factor (0.118) > land use factor (0.042) > rainfall factor (0.048) (Figure 6). The implementation of the GGP directly led to changes in the driving forces of land use and vegetation cover factors on soil erosion, and indirectly led to the alteration of the slope factor driving force. After that, the vegetation cover factor changed from the third driver to the dominant driver, the slope factor changed from the dominant driver to the second driver, the land use factor changed from the second driver to the third driver, and the rainfall factor driver did not change significantly. The dominant driving forces of the spatial distribution of soil erosion in the upstream, midstream, and downstream areas were transformed after the implementation of ecological engineering. Before that, the slope factor was the dominant driver in the upstream, midstream, and downstream areas, with driving forces of 0.446, 0.371, and 0.413, respectively. After that, the vegetation cover factor became the dominant driver, and the driving forces of the vegetation cover factor in the upstream, midstream, and downstream areas were 1.4 times, 2.7 times, and 1.9 times the slope factor, respectively. All soil erosion in the upstream, midstream, and downstream areas was affected by the GGP, but the magnitude of the influence was different, which was mainly caused by the varying intensities and strengths of the implementation of the project in different areas. The transformation of the vegetation coverage factor into a dominant driving force occurred in 2002, 2003, 2001, and 2002 for the entire watershed and the upstream, midstream, and downstream areas, respectively (Figure 7). Thus, it can be seen that the impact of the GGP on the spatial distribution of soil erosion in the Yanhe River Basin had a lag and temporal variability. This mainly occurred because ecological engineering was implemented at different times and scales in different regions, which was due to the influences of the natural conditions and socioeconomic status in the Yanhe River Basin.

#### 3.3.2. Changes in Natural Driving Force and Human Activity Driving Force before and after Implementation of GGP

**Figure 8:**
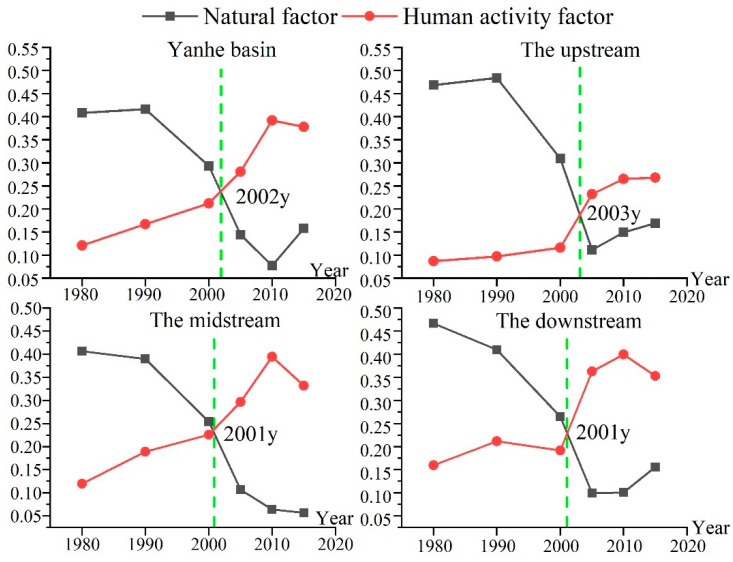
Driving forces of human activities and natural factors on the spatial differentiation of soil erosion.

From 1980 to 2015, the driving forces of natural factors on the spatial differentiation of soil erosion showed a decreasing trend in the entire Yanhe River Basin, while the driving forces of human activities showed an increasing trend (Figure 8). Before the implementation of the GGP, the natural factor driver was the dominant driver. However, the driving force of natural factors continued to decrease, and the driving force of human activities continued to increase, so the human activity factor driver exceeded the natural factor driver and became the dominant driver after the implementation of the GGP. Then, the average driving forces of anthropogenic factors in the upper, middle, and lower reaches were 0.220, 0.327, and 0.312, respectively, and the average driving forces of natural factors were 0.120, 0.185, and 0.155, respectively. In the upstream, midstream, and downstream regions, the human activity factors becoming the dominant driving force were not synchronized, with the earlier occurrence in the midstream and downstream regions occurring in 2001, and the later occurrence in the upstream region occurring in 2003. This may be related to the timing and scale of the implementation of the GGP and influenced by the natural background conditions of the Yanhe River Basin. The implementation of ecological engineering changed the spatial pattern of the soil erosion, which was originally controlled by natural conditions, and compensated for the constraints of poor natural background conditions on the development of the ecological environment. This research will have guiding significance for the implementation of soil and water conservation in loess hilly–gully areas in the future.

### 3.4. Changes in Interaction Driving Forces of Spatial Distribution of Soil Erosion before and after Implementation of GGP

#### 3.4.1. Detection of Interaction Driving Forces of Spatial Distribution of Soil Erosion

From 1980 to 2015, the interaction driving the soil erosion spatial distribution was mainly nonlinear enhancement, and only a small portion of the interaction was two-factor enhancement, which implied that the interaction of the two factors strengthened the soil erosion spatial distribution. Except for 1980, the driving force of slope ∩ vegetation coverage was the largest, reaching 0.467, and the values for each period were 0.415, 0.514, 0.407, 0.408, 0.516, and 0.540, respectively (as shown in Table 3). Except for 1980 and 2010, the driving force of land use ∩ rainfall was the smallest, at only 0.107, and was not very different in each period, at 0.140, 0.104, 0.116, 0.111, 0.042, and 0.128, respectively. The driving forces for land use ∩ slope, land use ∩ vegetation cover, rainfall ∩ slope, and rainfall ∩ vegetation cover were not significantly different at 0.267, 0.278, 0.275, and 0.256, respectively. The driving force of slope ∩ vegetation cover, as the first interaction driver, affected 40~50% of the soil erosion spatial distribution. This implies that when the slope remains constant as a topographic factor, the spatial distribution pattern of soil erosion can be altered by changing the vegetation cover. The results showed that the driving forces of slope ∩ land use and slope ∩ rainfall all showed decreasing trends, and the driving forces of vegetation cover ∩ land use and vegetation cover ∩ rainfall all showed increasing trends.

#### 3.4.2. Changes in Interaction Driving Forces before and after Implementation of GGP

Before the implementation of the GGP, the mean interactive driving force was slope ∩ vegetation cover > slope ∩ land use > slope ∩ rainfall > vegetation cover ∩ land use > vegetation cover ∩ rainfall > land use ∩ rainfall, and after that, it was vegetation cover ∩ slope > vegetation cover ∩ land use > vegetation cover ∩ rainfall > slope ∩ rainfall > slope ∩ land use > land use ∩ rainfall. Among the interactive drivers of the spatial differentiation of soil erosion, the dominant driving force was the interaction of slope and other factors before the implementation of the GGP, and it subsequently changed to the interaction of vegetation cover and other factors. After the implementation of ecological engineering, the driving forces of vegetation cover ∩ slope, vegetation cover ∩ land use, and vegetation cover ∩ rainfall sharply increased, mainly caused by the increase in vegetation cover. When the slope remained unchanged, the driving force of slope ∩ land use decreased sharply. This conclusion demonstrated that the GGP could directly increase the effects of vegetation cover on the spatial differentiation of the soil erosion and indirectly reduce the influence of slope on soil erosion by increasing the vegetation coverage.

## 4. Discussion

### 4.1. Differences in Effects of Rainfall Factors on Temporal and Spatial Variations in Soil Erosion

From 1980 to 2015, the main driving forces of the spatial distribution of soil erosion in the Yanhe River Basin were the slope factor and the vegetation cover factor, and the rainfall factor driving force had the least influence on the spatial distribution of soil erosion in the Yanhe River Basin, which differed from the conclusion that the rainfall factor was an important driving force for the temporal evolution of soil erosion in this area. This is mainly because geological and geomorphological conditions change slowly, and the alteration in rainfall intensity in the same area is much larger than the changes in topography and geomorphology over a short time period; thus, the influence of rainfall on the temporal evolution of soil erosion is larger than those of geological and geomorphological factors. The spatial gradient variation in the meteorological and geomorphological factors has a scale effect. In the loess gully and hilly area, the gradient change in geomorphological conditions is larger than that of the rainfall gradient when the study scale is small, so the influence of geomorphological conditions on the spatial distribution of soil erosion is larger than that of rainfall.

### 4.2. Effect of GGP on Spatial Distribution of Soil Erosion

Before the implementation of the GGP, the slope factor was the leading driving force of the spatial differentiation of soil erosion in the Yanhe River Basin, which was consistent with the conclusion of Xu Jiongxin, Liu Zhongyi, and other scholars, and was mainly determined by the geological and geomorphological characteristics of the loess hilly–gully region [83,84] (pp. 94–98, pp. 55–60). After the implementation of the GGP, the vegetation cover factor became the dominant driver of the spatial variation in soil erosion in the Yanhe River Basin, which is consistent with the conclusions regarding the role of vegetation cover on soil erosion in the loess hilly–gully area reported by Liu Xiaoyan, Dang Suzhen, Dong Zhengkai, Mu Xingmin, and Yang Shengtian [31,85] (pp. 505–518, pp. 135–141). It was shown that the implementation of ecological engineering caused a shift from cropland to woodland, high-cover grassland, and medium-cover grassland; from medium-cover grassland to woodland and high-cover grassland; and from low-cover grassland to woodland, high-cover grassland, and medium-cover grassland in the Yanhe River Basin [86] (pp. 15–21). The increase in vegetation cover and the change in pattern inevitably led to the change in the spatial pattern of soil erosion. After the implementation of the project, the magnitude and spatial distribution pattern of the slope remained unchanged; however, the driving force of the slope factor decreased significantly. It is worth considering whether this decrease in the spatial distribution of soil erosion driven by slope was caused by the GGP. By detecting the interaction driving force between vegetation cover and slope, it was further shown that although the GGP could not change the magnitude or spatial distribution pattern of the slope, it could indirectly reduce the driving force of slope on the spatial distribution of soil erosion by increasing the vegetation cover. After the implementation of the GGP, the average driving force of the land use factor decreased from 0.079 to 0.042. Considering the influence of newly cultivated sloping land, the average driving force of the conversion of sloping land to forestland or grassland on the spatial distribution of soil erosion was at least 3.7%. The average driving force of the vegetation cover factor increased from 0.066 to 0.273. Considering the damage to forestland and grassland, the average driving force of increasing vegetation cover of forestland and grassland on the spatial distribution of soil erosion was at least 20.7%. The average driving force of the slope factor decreased from 0.377 to 0.118. Considering the decrease in vegetation cover and resulting increased effect of slope on soil erosion, the average indirect driving force of the slope factor on the spatial distribution of soil erosion was at least 25.9%, which was caused by the increase in vegetation cover. Therefore, the average direct/indirect driving force of the GGP on the spatial distribution of soil erosion was at least 50.3%, and the actual average driving force may be greater than 50.3%. The effect of the implementation of ecological engineering was also influenced by the slope as well as natural conditions, such as rainfall, and the effect of natural factors on the spatial distribution of the soil may also be greater than the role in this study. This further inspires us to make full use of the first law of geography and to use connected and systematic thinking to explore and solve problems in depth.

### 4.3. Limitations and Prospects

The main insights from this study are that ecological engineering measures can improve poor natural conditions to a certain extent, while the implementation effect of ecological engineering is also limited by topography, rainfall, and other natural conditions; thus, the consideration of natural suitability should be emphasized in the ecological management process. Topography is the most fundamental driving force of the spatial differentiation of soil erosion in loess hills and ravines. Ecological engineering can weaken the influence of topography on soil erosion by improving vegetation cover, and the effect of soil and water conservation is significant. There is a general consensus among academics that thousands of years of human activities have severely damaged the vegetation conditions of the LP, leading to increased soil erosion in the region; however, we have overlooked the fact that human activities have also profoundly changed the topographic and geomorphic conditions of the LP, which has had a deep-rooted effect on soil erosion and is difficult to reverse by the actions of human activities. There is a “qualitative” difference between the current LP area and the area from thousands of years ago. The ecological balance of the previous LP area involved many aspects that are beyond the reach of the current LP. Therefore, the management of the LP must take into account the natural and socioeconomic conditions at this stage, and the most important task is to consider human society as an important subsystem of the ecosystem.

On the one hand, due to the limitations of data and information, in this study, the selection of the driving factors for the spatial differentiation of soil erosion mainly referred to the existing research results of domestic and foreign scholars. We selected only the main factors that affect soil erosion in the loess hilly and gully area, so the results may only be applicable to the Loess hills and gully landform area. In addition, vegetation coverage is a key indicator of this research, and it was mainly obtained through the calculation of the *NDVI*. The *NDVI* data before 2000 used a resolution of 8 km, whereas a 250 m resolution was used since 2000. The difference in the resolution of the *NDVI* data before and after 2000 would have a certain impact on the research results. However, in the current research, this problem basically exists in the use of all *NDVI* data, and some scholars have compared and analyzed the two resolutions of *NDVI* data and concluded that the effect of the difference in the *NDVI* resolution on vegetation cover is within an acceptable error range. Therefore, it is reasonable to use *NDVI* data with two different resolutions in this study.

## 5. Conclusions

Based on “3S” technology, the RUSLE model, and the GeoDetector model, this study quantitatively evaluated the effects of the GGP on soil erosion spatial differentiation in the Yanhe River Basin, a typical region of the LP. The study showed that the implementation of ecological engineering changed the dominant driving force of soil erosion spatial differentiation from the slope factor to the vegetation cover factor, and it changed the dominant interaction driving force between the slope factor and other factors to between the vegetation cover factor and other factors, resulting in a significant weakening trend of soil erosion. The detection of the interactive driving force of the spatial differentiation of soil erosion by the GeoDetector model quantitatively revealed the intricate relationship between the influencing factors of soil erosion. The increase in vegetation coverage caused by ecological engineering had both direct and indirect effects on soil erosion, which contributed 50% to the spatial differentiation of soil erosion. By quantitatively evaluating the direct and indirect effects of the GGP on soil and water conservation on the LP, the results further demonstrated that ecological engineering can effectively inhibit the water soil erosion on the LP. It also enlightens us that during the implementation of soil and water conservation in the future, we should consider the interaction between various influencing factors and take measures according to local conditions.

## Figures and Tables

**Table 1:** Assignment table of the farming measure factor *P*.

Slope range	≤5°	5°–10°	10°–15°	15°–20°	20°–25°	θ > 25°
*p* value	0.100	0.221	0.305	0.575	0.705	0.800

Comment: Report on the research results of soil erosion prediction model development project on northwest LP, Monitoring Center of Soil and Water Conservation of Ministry of Water Resources.

**Table 2:** Evaluation table of interaction detection results.

Reference for Judging	Interaction
*q*(*X*_1_ ∩ *X*_2_) < Min(*q*(*X*_1_),*q*(*X*_2_))	Nonlinear reduction
Min(*q*(*X*_1_),*q*(*X*_2_)) < *q*(*X*_1_ ∩ *X*_2_) < Max(*q*(*X*_1_),*q*(*X*_2_))	Single factor nonlinear reduction
*q*(*X*_1_ ∩ *X*_2_) > Max(*q*(*X*_1_),*q*(*X*_2_))	Two-factor enhancement
*q*(*X*_1_ ∩ *X*_2_) = *q*(*X*_1_) + *q*(*X*_2_)	Independence
*q*(*X*_1_ ∩ *X*_2_) > *q*(*X*_1_) + *q*(*X*_2_)	Nonlinear enhancement

**Table 3:** Interactive detection results of the driving forces for the spatial distribution of soil erosion.

	A ∩ B	C = A ∩ B	D = A + B	Judgment	Interactive
1980	lucc ∩ slo	0.404	0.426	A ∩ B < A + B	↑
	lucc ∩ vfc	0.132	0.121	A ∩ B > A + B	↑↑
	lucc ∩ pre	0.140	0.129	A ∩ B > A + B	↑↑
	slo ∩ vfc	0.415	0.400	A ∩ B > A + B	↑↑
	slo ∩ pre	0.432	0.408	A ∩ B > A + B	↑↑
	vfc ∩ pre	0.174	0.104	A ∩ B > A + B	↑↑
1990	lucc ∩ slo	0.463	0.485	A ∩ B < A + B	↑
	lucc ∩ vfc	0.176	0.167	A ∩ B > A + B	↑↑
	lucc ∩ pre	0.104	0.096	A ∩ B > A + B	↑↑
	slo ∩ vfc	0.514	0.488	A ∩ B > A + B	↑↑
	slo ∩ pre	0.419	0.416	A ∩ B > A + B	↑↑
	vfc ∩ pre	0.108	0.099	A ∩ B > A + B	↑↑
2000	lucc ∩ slo	0.339	0.349	A ∩ B < A + B	↑
	lucc ∩ vfc	0.213	0.212	A ∩ B > A + B	↑↑
	lucc ∩ pre	0.116	0.120	A ∩ B < A + B	↑
	slo ∩ vfc	0.407	0.385	A ∩ B > A + B	↑↑
	slo ∩ pre	0.299	0.293	A ∩ B > A + B	↑↑
	vfc ∩ pre	0.133	0.157	A ∩ B < A + B	↑
2005	lucc ∩ slo	0.110	0.117	A ∩ B < A + B	↑
	lucc ∩ vfc	0.300	0.281	A ∩ B > A + B	↑↑
	lucc ∩ pre	0.080	0.082	A ∩ B < A + B	↑
	slo ∩ vfc	0.408	0.342	A ∩ B > A + B	↑↑
	slo ∩ pre	0.152	0.144	A ∩ B > A + B	↑↑
	vfc ∩ pre	0.267	0.308	A ∩ B < A + B	↑
2010	lucc ∩ slo	0.095	0.099	A ∩ B < A + B	↑
	lucc ∩ vfc	0.420	0.392	A ∩ B > A + B	↑↑
	lucc ∩ pre	0.042	0.040	A ∩ B > A + B	↑↑
	slo ∩ vfc	0.516	0.429	A ∩ B > A + B	↑↑
	slo ∩ pre	0.081	0.077	A ∩ B > A + B	↑↑
	vfc ∩ pre	0.377	0.370	A ∩ B > A + B	↑↑
2015	lucc ∩ slo	0.082	0.085	A ∩ B < A + B	↑
	lucc ∩ vfc	0.410	0.378	A ∩ B > A + B	↑↑
	lucc ∩ pre	0.128	0.117	A ∩ B > A + B	↑↑
	slo ∩ vfc	0.540	0.418	A ∩ B > A + B	↑↑
	slo ∩ pre	0.183	0.158	A ∩ B > A + B	↑↑
	vfc ∩ pre	0.443	0.451	A ∩ B < A + B	↑

Comment: *A* and *B* represent driving factors *X*_1_ and *X*_2_ of the spatial distribution of soil erosion, respectively; *A* ∩ *B* represents the value of the interaction driving force between driving factors *X*_1_ and *X*_2_, *q*(*X*_1_ ∩ *X*_2_); *A* + *B* represents the sum of driving force *q*(*X*_1_) and *q*(*X*_2_); and “↑” and “↑↑” imply two-factor enhancement and nonlinear enhancement, respectively.

## Data Availability

Most of the datasets used and/or analyzed during the current study are available from the corresponding author on reasonable request.
